# The Health Status of Informal Waste Collectors in Korea

**DOI:** 10.3390/ijerph17155363

**Published:** 2020-07-25

**Authors:** Joonho Ahn, Jaeyong Lee, Hyeyeon Park, Yangwon Kang, Chungwon Kang, Young-Jin You, Mo-Yeol Kang

**Affiliations:** 1 Department of Occupational and Environmental Medicine, Seoul St. Mary’s Hospital, College of Medicine, The Catholic University of Korea, Seoul 06591, Korea; drcox@naver.com (J.A.); monsep86@naver.com (J.L.); 2 Department of Psychology, California State University, Long Beach, CA 90840, USA; dus0325@nate.com; 3 Department of Occupational and Environmental Medicine, Korea Workers’ Compensation & Welfare Service Ansan Hospital, Ansan 15324, Korea; yw.kang.green@gmail.com; 4 Department of Preventive Medicine, College of Medicine, Seoul National University, Seoul 03080, Korea; scmfer@hanmail.net; 5 Center for Occupational and Environmental Medicine, College of Medicine, The Catholic University of Korea, Seoul 06591, Korea; frenzyjin@hanmail.net

**Keywords:** informal waste collectors, occupational injury, workplace risk factors, depression, musculoskeletal pain

## Abstract

*Background*: A broad, holistic approach was performed among informal waste collectors (IWCs) in Korea to understand their complex multidimensional health and safety problems. *Methods*: In the quantitative study, a survey of IWCs was conducted at four junk shops in Gangbuk-gu, Seoul, and survey data were used to calculate age-standardized prevalence rates based on comparisons with the general population in Korea. A qualitative study was also performed to provide more details on IWCs’ occupational and musculoskeletal injuries and depression. *Results*: In the quantitative study, the age-standardized prevalence rate (aSPR) of occupational injury was higher than that of the general standard population (aSPR: 10.42, 95% confidence interval (CI) 5.19–18.64) and that of blue-collar workers (aSPR: 4.65, 95% CI 2.32–8.32). Regarding musculoskeletal problems, compared to employed populations, the aSPRs of shoulder pain (aSPR: 2.63, 95% CI 1.60–4.06), wrist pain (aSPR: 3.33, 95% CI 1.33–6.86), knee pain (aSPR: 1.51, 95% CI 1.01–2.17), and ankle pain (aSPR: 3.54, 95% CI 1.14–8.26) were higher. Regarding psychological problems, depression (aSPR: 2.55, 95% CI 1.27–4.56) and suicidal or self-harm ideation (aSPR: 2.09, 95% CI 1.11–3.58) were higher compared to general populations. Through the qualitative study and case study on muscular problems, more details on the work environment problems of IWCs were obtained. *Conclusions*: IWCs are exposed to various occupational hazards and lack proper protection. They show a high prevalence of occupational injury, musculoskeletal disease, and depression.

## 1. Introduction

Global waste generation rates are rising quickly due to increasing populations and economic growth. According to the World Bank’s What a Waste 2.0 report, the world generates 2.01 billion metric tons of municipal solid waste annually, and rapid urbanization, population growth, and economic development will increase global waste by 70% over the next 30 years to a staggering 3.40 billion annual metric tons of waste [1]. Globally, it is estimated that 50 million people work in dangerous and unhygienic conditions collecting, sorting, and disposing of waste. A significant portion of solid waste is collected by informal waste collectors (IWCs), of which there are estimated to be more than 15 million, globally. IWCs are typically women, children, the elderly, migrants, and those who would otherwise be unemployed. In South Korea, the elderly and people with low incomes have also entered this market; their numbers are around 2 million [2].

**Figure 1:**
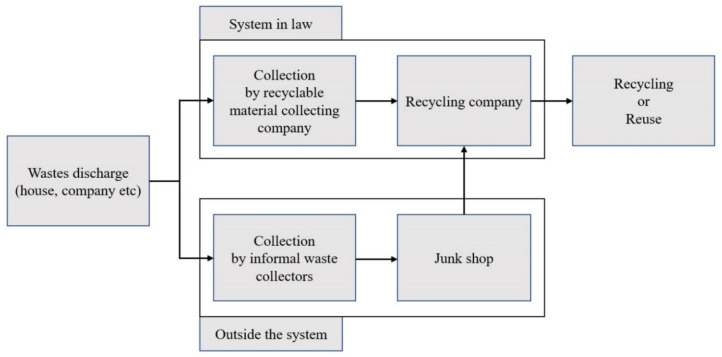
Recycling process in Korea and the role of informal waste collectors.

Comparative cost-efficiency is the reason that such a large share of recyclables is collected by IWCs (Figure 1), who operate in the grey economy, which lacks occupational and safety standards. IWCs serve as a valuable link in the waste management chain, providing inexpensive materials for the next level in the chain. This “work” is generally performed outside legal and institutional frameworks, but it greatly contributes to the 20–50% recycling rate in the waste management industry [3]. In spite of their contributions, society does not fully recognize the role and value of IWCs.

Informal waste collection (“informal recycling”), a ubiquitous activity, is defined as the collection, separation, sorting, and selling of solid waste as a means for individuals to maintain or supplement income. Informal recycling can be an income-generating strategy for individuals with low or no income. It involves the collection and sale of recyclable and reusable items from public waste and curb waste from households and businesses [4]. In South Korea, most types of paper can be returned through a recoverable deposit system, which provides a significant income opportunity for IWCs. In Seoul, the capital of South Korea, some IWCs work throughout the city, focusing on specific areas or parks, whereas others rely on regular routes and partnerships with residences and businesses to optimize their material recovery efficiency [5].

The work of IWCs is not only unstable but also unofficial due to its untaxed and unregulated status in urban and political economies. Because handling waste and recycling materials is work that is not usually protected by regulations, it is often performed in poor working conditions [6]. Consequently, IWCs are exposed to many different types of hazards, including physical, ergonomic, chemical, biological, psychosocial, environmental, and other safety hazards. These exposures can result in serious health problems.

While informal waste collection is still a widespread practice, even in developed countries, there is a noticeable lack of data on the health status of IWCs. Thus, policy makers have limited information on which to base improvements in working conditions. Against this backdrop, this study was designed to investigate IWCs’ major health and safety issues, identify the causes of these issues, and formulate measures to reduce inherent risks. A comprehensive and holistic approach was needed to explore the complex and multidimensional nature of IWCs’ physical and mental health challenges. Hence, this study used a mixed quantitative and qualitative approach.

## 2. Methods

### 2.1. Study Design

**Figure 2:**
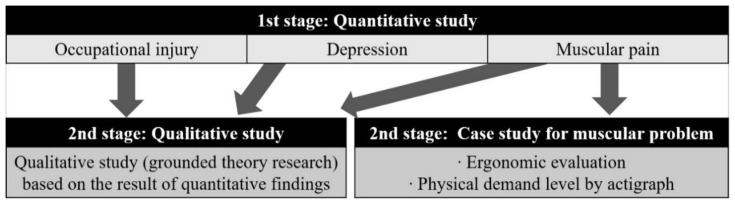
Study model of the health and safety problems amongst informal waste collectors.

To assess the health and safety of the IWCs, this study first examined the standardized prevalence of occupational injury, musculoskeletal pain, and depression (Figure 2). To investigate the causes underlying the quantitative research results, a qualitative study and a case study on muscular problems were conducted.

### 2.2. Quantitative Study and Statistical Analyses

Among the 17 junk shops in Gangbuk-gu, Seoul, 4 shops participated in the study. A survey was conducted at four shops, where owners gave permission for the survey, which had the necessary space. Participants were IWCs who collected trash at least once per week, who agreed to take the survey between 1 August and 31 October 2019, at one of the four junk shops. Duplicate questionnaires and questionnaires of individuals who delivered trash to junk shops but were not involved in its collection were excluded from the study. There were 54 final participants, but one participant did not provide a date of birth. Thus, 53 people ultimately participated in the age standardization analysis.

The age-standardized prevalence rates (aSPRs) were calculated for IWCs and compared to those of the general population in Korea. The rates were age-standardized using the indirect method of standardization [7], and 95% confidence intervals (95% CI) were calculated, according to Byar’s approximation [8]. Age standardization was performed using survey data to provide estimations for the population in Korea. For each variable, a question from a national survey was used to compare IWCs with the external population. The Korea Health Panel (KHP), Korea National Health and Nutrition Examination Survey (KNHANES), and the Korean Longitudinal Study of Ageing (KLoSA), are nationally representative datasets of the Korean general population, and the all study designs are stratified, multi-stage cluster samples. From the KHP 2016, a question about visits to the emergency room was used in cases of injuries over the past year, as well as a question about locations where injuries occurred, such as a workplace. From the KNHANES 2016, the Patient Health Questionnaire-9 (PHQ-9), which assesses depression symptoms during the previous 2 weeks, was used. From the KLoSA2016, a questionnaire to assess current musculoskeletal pain was used. The KLoSA survey includes broad questions about pain sites. Participants were asked to estimate their pain location by answering the following question: “At which location are you experiencing pain now? Please check all: shoulder, arm, wrist, low back, leg, knee, ankle.” The case of answering “yes” to each question was defined as musculoskeletal disorder for each site. For sensitivity analysis, various comparisons were performed, and the general population, the employed, the unemployed, and blue-collar workers were used as the reference population. Statistical analysis was conducted for the health and safety status prevalence of the recyclable material collectors and the reference population using SAS, version 9.4 (SAS Institute, Cary, NC, USA), and the aSPRs of health and safety status were calculated using Microsoft Excel.

### 2.3. Qualitative Study

A qualitative study was conducted to obtain more detailed information on IWCs’ depression and occupational and musculoskeletal injuries. The approach was based on a sequential explanatory mixed methods design, as proposed by Creswell and Plano-Clark [9].

Five individuals were selected for the sample (Table 1). Based on author judgment, two men and two women who had sufficient experience collecting waste were recruited, and the owner of a junkyard who had close contact with the IWCs was included as well. The non-governmental organization (NGO), Beautiful Life Love, which provided support to IWCs in the past, helped to recruit the participants. The participants’ in-depth interviews took place from October to December 2019, and all interviews used open questions. Questions were asked until the point of data saturation, and each participant was interviewed two or three times. Before the in-depth interviews, the study’s purpose, overall outline, and main questions were verbally explained to participants. In addition to requesting participants’ permission for the interview process and records, written consents were obtained. In-depth interviews were conducted on the theme of waste collection work. Examples of questions are as follows: “How do occupational injuries occur during waste collection?”, “Why are rates of depression and suicide high in IWCs?”, and “What causes the muscular pain of the IWCs?”

To analyze the data, grounded theory techniques were used. Grounded theory is particularly useful when little is known about participants and an appropriate theory does not exist to explain or predict group behavior [10]. In the grounded theory process, data analysis is based on coding [11]. In the first stage, open coding involves “the process of breaking down, examining, comparing, conceptualizing, and categorizing data” [12]. The second step involves axial coding, which is “a set of procedures whereby data are put back together in new ways after open coding, by making connections between categories.” That is, categories are connected using a paradigm model. The final step was to integrate the categories and describe the central category.

To ensure the reliability of the qualitative study, there were four things to consider [13]. First, regarding the truth value, the interviews were conducted in a relaxed atmosphere to facilitate the establishment of trust, and participants were encouraged to talk freely about their experiences. During an interview, participants’ previous statements were read aloud to ensure that the information was recorded correctly. Second, regarding applicability, a participant would confirm what he or she reported in the previous interview and was then given an opportunity to add to that information. Third, for consistency, the entire process of the qualitative study was recorded in detail. The theme and subthemes were incorporated into tables, and quotes from the interviews were inserted where relevant. Finally, to maintain a neutral position, the author made prejudices and stereotypes transparent before data collection, and the researchers avoided making any prejudgments about participants.

### 2.4. Case Study on Muscular Problems

The in-depth interviews were limited in their determinations of musculoskeletal pain causes; thus, case studies based on ergonomic evaluations were conducted. Among the group of qualitative research participants, two agreed to participate in a further analysis and were selected for the case studies. First, actigraph was used to measure the participants’ energy consumption and heart rate per hour. Actigraph is a device that recognizes human body movements on three axes and measures and records this physical activity by time zone. Although there is no standardized reference for energy consumption per hour, the results of similar studies were used as a reference [14,15]. One study reported the hourly energy consumption of office workers, car manufacturers, and formwork carpenters; these results were compared with the energy consumption results of participants in this study. In addition, this study measured participants’ heart rate while they were working. Although the heart rate itself may indicate the workload to some extent, the overwork index was used to present it more intuitively. The overwork index is calculated by dividing the actual working hours by the maximum allowable working hours, calculated by the relative heart rate. If the participant’s overwork number is greater than 1.0, this means he or she is overworked. Additionally, each participant’s entire workday was filmed with a video camcorder. The filmed work was divided into hours and the musculoskeletal burden of physical activity was evaluated using the rapid entire body assessment (REBA) method.

### 2.5. Ethics Statement

The original study was approved by the Catholic University of Korea Catholic Medical Center, Seoul St. Mary’s Hospital Institutional Review Board (Board Approval Number: KC19QESI0331). Every participant signed a consent form, and anonymity and confidentiality were assured.

## 3. Results

### 3.1. Quantitative Study

The analysis included 54 participants. The majority of workers (83.33%) were over 65 years old, revealing some characteristics of older workers (Table 2). Working periods were distributed evenly for workers with more than 10 years of experience and less than 10 years of experience. Mainly, hard carts and shopping carts were used to transport collected recyclable materials. In terms of the average collection weight, “less than 50 kg” was the most frequently cited at 53.7%, while 44.44% of workers reported “more than 50 kg.” Some workers used vehicles instead of carts, and these workers were able to transport an average weight of over 200 kg. Overall, 20.37% of workers collected recyclable materials once or twice a week, and notably, 48.15% of the workers collected recyclable materials every day.

In this study, 37.04% of participants had experienced occupational injury. Among those who were injured, 40% had injuries from car accidents and 60% had injuries from falling. Regarding treatment, among those who were injured, 16.67% had been hospitalized, 33.33% had outpatient treatment, 11.11% visited emergency rooms, and others were not treated in any medical setting.

Table 3 presents information on workers’ safety, musculoskeletal issues, and psychological problems. For participants treated for occupational injuries in hospitals, the aSPRs were calculated through comparisons with the general population, employed population, and blue-collar workers as the reference population. For participants in our study, the aSPR of occupational injury was higher than for all reference populations, even blue-collar workers. In terms of musculoskeletal problems, the prevalence of pain in the shoulder, lower back, and knee was high in our participants. However, compared to the reference populations, the aSPRs of shoulder, wrist, knee, and ankle pain were higher, but the aSPR for lower back pain was not. Regarding psychological problems, depression and suicidal or self-harm ideation were higher in our participants compared to most reference populations.

### 3.2. Qualitative Study

**Figure 3:**
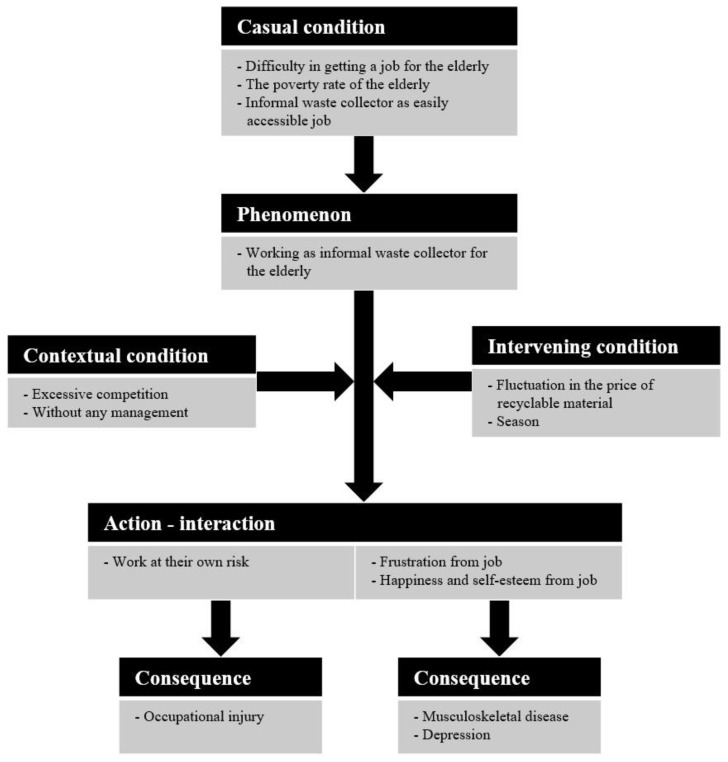
Conceptual model of themes and relationships.

Through open coding, 27 subthemes and 14 themes were derived (Table S1). The conceptual models (Figure 3) were organized into six broad categories (casual condition, phenomenon, contextual condition, intervening condition, action-interaction, and consequence) related to IWCs through the axis coding.

The categories corresponding to the causes of phenomena were linked to causal conditions. The causal conditions category was developed to capture the underlying causes, as perceived by participants in the three themes. “Poverty in the elderly” included participants’ descriptions of why they chose to engage in waste collecting, for example, because of family financial troubles or inadequate amounts from national pensions. When it is difficult for the elderly to find a job, it aggravates their poverty and they are induced to do work that is easily accessible.

The main theme in the phenomenon, “the elderly working as IWCs,” consisted of two subthemes. Some participants worked by collecting in the street. However, others had unofficial contracts with a store. They might be received by a store, exclusively, and help maintain cleanliness in that store. This phenomenon was linked with the contextual conditions of “excessive competition” and “without any management”. The competition among the IWCs is becoming increasingly intense, and a lack of management is commonly found in various situations. The intervening condition that changes the intensity of the phenomenon is “fluctuation in the price of the recyclable material” and “season”.

Participants’ actions or interactions with the phenomenon were “working at their own risk” in a physical sense and “social stigma, happiness, and self-esteem from job” in a psychological sense. These actions or interactions led to the occupational injury, muscular pain, and depression that were identified in quantitative studies.

Amidst severe competition, the reaction of IWCs appears to be to take more risks, such as by working in impoverished areas at night, engaging in dangerous collections around driveways, using poor working postures, and so on. In a psychological sense, they showed ambivalent responses that were both positive and negative. 

### 3.3. Case Study on Muscular Problems

Table 4 shows the daytime biometric results from actigraphy. The average hourly energy consumptions of the two participants were measured at 86.61 and 140.83 kcal, respectively. Comparing with previous studies [14,15], this consumption amount was 5.6 times the office worker’s energy consumption and 2.6 times a car manufacturer’s energy consumption (Table S2). A formwork carpenter had the most similar energy consumption to that of IWCs in this study. For the overwork index based on the relative heart rate, results showed that participant A was performing appropriate tasks with an index of 0.31, but participant B was overworked, with an index of 2.83.

The IWCs’ work consisted mainly of four tasks: collecting, carrying, classifying, and moving material. Each task was evaluated by means of the REBA method to assess the musculoskeletal risk level (Table S3). In the collecting task, a body position ratio of more than 50% (with an above-moderate risk) was observed in both participants. In the collecting task, 85.7% of participant B’s postures were at an above-moderate risk. In addition, in the carrying task, 57.1% of participant A’s postures and 33.3% of participant B’s postures were at moderate or higher risk. In contrast, 58.8–71.4% of postures were low risk for classifying tasks. These tasks were obviously less risky than the collecting and carrying tasks. Furthermore, the risk of most posture-moving tasks was normal. In the 1-day task assessment, conducted without distinctions between tasks, 29.8% of the postures of target A and 40.6% of the postures of target B were rated as above-moderate risk.

During the 1-day tasks, the postural risks were assessed according to each body part (Table S4). It was found that most postures put less pressure than expected on the neck and legs. Furthermore, it was found that there were minor to moderate burdens on lower arms and wrists, even though 56.8–62.5% of lower arm tasks were assessed as non-burdening and 64.1–75.7% of wrist tasks were assessed as non-burdening. On the contrary, the upper arms and trunks were found to be heavily burdened, especially trunks, since the proportion of straight posture (a non-burdening posture) was only 18.8% for participant A and 13.5% for participant B, while the remaining postures were mostly burdened. In the evaluation of the upper arm, “bent forward at 45–90°”, which is a moderately or highly burdened posture, accounted for 31.3–35.1% and “bent forward over 90°” accounted for 4.7–10.8%.

## 4. Discussion

To our knowledge, this is the first study on the health status of IWCs in the South Korean population. A quantitative study was conducted to identify the risks of injury, musculoskeletal pain, and depressive symptoms in IWCs. The results of the qualitative study shed light on the causes of these IWC health problems. In our conceptual model, “difficult for elderly to find jobs,” “poverty in the elderly”, and “IWC as an easily accessible job” were identified as the main motivations for the work of waste collecting. These phenomena were influenced by several conditions and worker actions, with health consequences, such as occupational injury, musculoskeletal disease, and depression.

These results are consistent with those of previous studies. Studies from many countries—Argentina, Brazil, the Philippines, Vietnam, and other countries in South Asia, as well as the U.S. and Canada—have highlighted that IWCs face a wide range of occupational health risks. These include the risks of musculoskeletal damage and other injuries, along with mental health challenges [16]. Most of these studies have applied a mixed methodology approach, utilizing standardized surveys, awareness-raising interviews and focus groups, researcher observations, and comparisons with national population referees. Occupational injuries common to IWCs are fractures, falls, lacerations, blunt trauma, and traffic accidents [16]. Traffic accidents were shown to be a high perceived risk for IWCs, but IWCs themselves have some control over this risk. They can increase their risk of accidents by violating traffic rules, such as by jay walking, which is considered a high-risk behavior in older individuals [17]. Most IWCs do not use luminous vests or stickers, making it difficult for drivers to see them and increasing the risk of accidents [18]. In South Korea, the majority of IWCs begin work at dawn and continue until after nightfall, which means they are working in dark streets. Furthermore, IWCs often prefer to work with bare hands to increase their tactile receptivity; however, working this way could be dangerous. Waste sometimes includes glass, sharp building materials, or hospital garbage, such as syringes. In the summer, IWCs might wear shorts and t-shirts, which provide little to no protection for arms or legs.

The high rate of musculoskeletal pain in IWCs is not surprising, considering their work patterns. Their musculoskeletal problems are direct results of ergonometric stress from repetitive movements and lifting heavy objects. These activity patterns can lead to fatigue, pain, sprains, or strains, and other musculoskeletal disorders. A study in Brazil found that waste collecting tends to involve squatting, vibrations, awkward postures, and repetitive movements. Frequent kneeling occurs during the sorting and collection of paper and is often associated with limb pain. In this study, IWCs showed excessive physical activity, with average hourly energy consumptions similar to that of formwork carpenters. In addition, IWCs often assumed uncomfortable postures while at work.

Regarding depression, a lack of financial security, along with shame and being socially marginalized, lead to higher self-rated psychological vulnerabilities [16]. IWCs reported constant occupational threats from injury, harassment, or bullying. Undoubtedly, multiple work hazards, stigma, and a lack of financial security would aggravate stress in any workers. In South Korea, as well as in many other countries, street cleaning and garbage collection are seen as a “3D” (dangerous, dirty, and difficult) job. Under these circumstances, workers are bound to have lower self-esteem. The IWCs interviewed in this study explicitly linked their experience of poverty with insufficient social safety nets (e.g., income assistance, national pensions, disability payments).

To reduce the health risks of IWCs, we believe the following measures need to be taken. First, IWCs should be given proper education on minimizing work risks and using proper hygiene. A lack of safety training may be a major factor contributing to their workplace injuries. In particular, to reduce traffic accidents, it is essential to encourage IWCs to follow traffic rules. Job-specific guidelines that include safety measures could help IWCs cope with risks inherent in their jobs. To this end, local public social welfare institutions could be used as a channel for education. These institutions could provide training on the safe use of manual carts and demonstrate exercises and stretches to prevent and mitigate musculoskeletal injuries. Furthermore, training could be provided on minimizing musculoskeletal burdens, using correct postures and proper lifting and carrying methods. The institutions could provide equipment, such as waist protectors, especially for those who are already experiencing symptoms. This could prevent deterioration into acute symptoms.

Second, ergonomic interventions are needed to prevent musculoskeletal disorders. The use of hand carts made of lighter materials, such as hardened plastics, should be encouraged. Additionally, IWCs could be encouraged to use carts with larger wheels, given that the roads they use may be in poor condition. Moreover, to prevent occupational injuries at night, luminous stickers could be attached to the backs of cars and luminous vests could be distributed. As the workers could lose these vests, periodic replacement would be preferable to one-time distribution.

Third, an elderly welfare support system should be actively implemented for IWCs. Along these lines, increasing investments in health outreach and providing sanitation and first aid services are also warranted. In addition, because IWCs have few chances to form interpersonal relationships, and thus suffer an increased risk of depression, various linkage programs (e.g., visiting programs) should be developed in the community. Social welfare facilities should strive to enhance the life of the elderly through social, physical, and psychological support. Lastly, given the fact that IWCs mostly belong to a socioeconomically marginalized and vulnerable group, financial support measures should be made available that are based on realistic standards for the elderly in dire circumstances.

This study had some limitations. First, our research was limited to Gangbuk-gu in South Korea. Therefore, generalization may be difficult. Second, the health outcomes reported in this study are based on self-reporting, which may be a limited mechanism. When respondents are assessing their own health, they may make errors or false statements based on social desirability. Moreover, we could not adopt structured or comprehensive instruments to measure psychosocial risks of the work. We obtained information regarding that only from the interview. This could limit the assessment of social determinants for IWC’s health. Third, quantitative research was conducted only with the prevalence rate of standardization, and adjustments for confounding factors were not implemented. Fourth, because this is a cross-sectional study, the disadvantage is that causal relationships are difficult to determine. In particular, it is difficult to conclude the question as to whether the work of IWCs causes the indicated ailments or rather the possession of these ailments disqualifying from other jobs condemns them to informal waste collecting. To this end, a future longitudinal study is needed.

In conclusion, it was found that IWCs are exposed to occupational hazards, some of which they cannot control. They showed a high prevalence of occupational injuries, musculoskeletal disorders, and depression. Therefore, we conclude that this group should be treated as a vulnerable group in need of special care. We hope that this study provides an overview for city planners, community groups, and social researchers to improve the health and social life of IWCs through effective government ordinances, basic health services, community development, welfare programs, and public policies. However, our investigation was limited to a specific population in South Korea, and thus it allows for only tentative conclusions. Further studies should include different methodologies and information about other hazards, such as chemical and biological exposure. It would be valuable to determine which of the factors have the largest share in injuries or perceptible work difficulties. For example, future participatory studies could identify additional hazards and risks, enhancing the understanding of problems and informing future solutions.

## Figures and Tables

**Table 1:** General characteristics of study participants.

ID ^†^	Age	Sex	Recyclable Materials Collection	Working Period (Year)	Transportation	Previous Job	Participation ^‡^
1	72	male	working now.	15	bicycle	taxi driver	O
2	58	male	working now.	15	Rear car	cabinetmaker	O
3	76	female	She stopped working because of a stroke 4 years ago.	20	Baby carriage or shopping cart	none	X
4	85	female	working now.	15	Baby carriage or shopping cart	chef	X
5	45	female	junk shop owner for 20 years	0	none	non	X

^†^ participant identification; ^‡^ participation in case study for muscular problem.

**Table 2:** Basic characteristics of study population.

		*N*	%
Total		54	100.00
Gender			
	Male	24	44.44
	Female	30	55.56
Age (year)			
	51–60	6	11.11
	61–70	9	16.67
	71–80	24	44.44
	81 or over	14	25.93
	No response	1	1.85
Working period (year)			
	0–9	32	59.26
	10–19	17	31.48
	20–29	3	5.56
	30–39	2	3.7
Transportation			
	Rear car	24	44.44
	Baby carriage	2	3.7
	Shopping cart	17	31.48
	Bicycle	3	5.56
	Vehicle and motorcycle	5	9.26
	Other transport	3	5.56
Average collection weight (kg) when visiting junk shop			
	0–49	29	53.70
	50–99	12	22.22
	100–149	6	11.11
	150–199	1	1.85
	200-	5	9.26
	No response	1	1.85
Weekly Average Collection Days (days per a week)			
	1–2	11	20.37
	3–4	10	18.52
	5–6	6	11.11
	7	26	48.15
	No response	1	1.85

**Table 3:** Age-standardized prevalence rate (aSPR) for informal waste collectors’ safety, musculoskeletal, and psychological problems.

		Study Participants		aSPR ^†^ for the Standard Population		
		*N*	%	General Population	Employed Population	Blue-Collar Workers or Unemployed Population ^‡^
Total		54	100			
Safety problem						
	Occupational injury	20	37.04	**10.42 (5.19–8.64)** *	**5.04 (2.51–9.02)**	**4.65 (2.32–8.32)**
Musculoskeletal problem						
	Shoulder	20	37.04	**2.52 (1.54–3.90)**	**2.63 (1.60–4.06)**	**3.14 (1.92–4.85)**
	Arm	7	12.96	1.55 (0.62–3.19)	1.86 (0.74–3.83)	2.12 (0.85–4.36)
	Wrist	7	12.96	**2.49 (1.00–5.13)**	**3.33 (1.33–6.86)**	2.44 (0.98–5.02)
	Low back	22	40.74	1.01 (0.64–1.54)	1.05 (0.66–1.60)	1.06 (0.67–1.61)
	Leg	13	24.07	0.69 (0.37–1.18)	0.82 (0.44–1.41)	0.99 (0.53–1.69)
	Knee	29	53.70	1.34 (0.90–1.92)	**1.54 (1.03–2.21)**	**1.51 (1.01–2.17)**
	Ankle	5	9.26	2.65 (0.86–6.19)	**3.54 (1.14–8.26)**	**4.58 (1.48–10.68)**
Psychological problem ^§^						
	Depression ^¶^	11	21.15	**2.55 (1.27–4.56)**	**4.72 (2.35–8.45)**	**2.08 (1.04–3.73)**
	Suicidal or self-injury idea ^#^	13	25.00	**2.09 (1.11–3.58)**	**2.53 (1.35–4.33)**	1.86 (0.99–3.18)

^†^ aSPR: age-standardized prevalence rate. ^‡^ For safety and musculoskeletal problems, the blue-collar population was used as the standard population. For psychological problems, the unemployed population was used as the standard population. ^§^ No respondent: 2, total 52. ^¶^ Patient Health Questionnaire-9 (PHQ-9) score, within the last 2 weeks, 10 or more points. ^#^ Question 9 on Patient Health Questionnaire-9 (PHQ-9), more than 1 day within the last 2 weeks.* Bold entries denote parameter estimates significant at the 5% level.

**Table 4:** Energy consumption and heart rate related result per individual working hour.

	ID ^†^	
	A	B
Energy consumption per individual working hour (kcal)	86.61	140.83
Actual working time (hours)	2.50	10.00
Relative heart rate (%)	24.21	41.61
Maximum acceptable work time (hours)	8.15	3.53
Overwork index	0.31	2.83

^†^ participant identification.

## Supplementary Materials

The following are available online at https://www.mdpi.com/1660-4601/17/15/5363/s1, Table S1: Exemplary quotes, subtheme, theme, and broad categories; Table S2: Average hourly energy consumption by occupation; Table S3: Risk grade distribution by elements of work, according to REBA; Table S4: The distribution of body position.
