# Supplementary material for: The Health Status of Informal Waste Collectors in Korea
Source: Int J Environ Res Public Health. 2020 Jul 25;17(15):5363. doi: 10.3390/ijerph17155363 (PMC7432789; doi:10.3390/ijerph17155363)
Supplement: Supplementary file 1 [file ijerph-17-05363-s001.pdf]

International Journal of
Environmental Research and Public Health

MDPI

Supplementary Material:

Table S1. Exemplary quotes, subtheme, theme and broad categories.

Broad category	Theme	Subtheme	Exemplary quotes
Causal condition	The poverty of the elderly	Small amount of national pension	"Even if I am a basic livelihood security recipient with disability benefits, I get only 400,000 won a month. This money is tight for rent and living expenses." - Participant 1<br>"My family sent my husband to a nursing home because of his dementia. But I pay the hospital fee myself. National pension is not enough to us" - Participant 2
		Family trouble	"My wife divorced with me and died, and it's hard for me to ask my children for help." - Participant 1
	Difficulty in getting a job for the elderly	Era of unemployment crisis	"It's worse nowadays. Even young people have nothing to do, certainly old people have nothing to do." - Participant 1<br>"In this era of unemployment crisis, It's hard for my sons to get a job these days. Of course, the old men are barely able to get a job." - Participant 3
		Difficulty in learning new work	"When I quit the taxi, there was nothing to do." - Participant 1<br>"Informal waste collectors is the only thing a person who is sick like me and has learned nothing can do. " - Participant 2
	Easily accessible job (junk collector)	No job requirement for junk collector	"Anyone can do it(collect junk). I don't have to be allowed to pick up junk. We just have to go out and pick up junk " - Participant 1
		Neighbor's help	"Once upon a time, neighbors gathered old clothes and gave me"- Participant 3<br>"Once I pick it up, rather, people ask me to take waste." - Participant 4
Phenomenon	Working as a junk collector for the elderly	Collection in the street	"I'm dragging the rear car and picking up the recyclable materials on the street."- Participant 2
		Informal contracts with shops	"At the welfare center, I go to the same time every day and pick up recycled materials. Instead, welfare center workers don't let others take them. I have to be on time." - Participant 1<br>"To get more boxes, I worked at a vegetable store and got boxes." - Participant 3
Contextual condition	Excessive competition	Participation of many people	"The competition is intense. This neighborhood is so poor that everybody picks up junk. I've fought with others because of junk. Some people lied that junk was theirs." - Participant 1<br>" Ugh. This is not a rich neighborhood. I think old people in this neighborhood almost do this when the national economy is difficult." - Participant 5
	Without any management	Without any management	"This job is not someone who leads me to work. This job is what I want to do" - Participant 3
Intervening condition	Fluctuation in the price of recyclable material.	Fluctuation in the price of recyclable material.	"The price of waste paper is finally decided by the paper companies. But it is affected by the import / export volume. So the price change is severe. At the beginning of the year, the price of waste paper was 70-80 won/kg, but now it's only 30 won/kg. At this time, elderly people hardly come to junk shop. On the contrary, when prices rise, they come a lot." - Participant 5<br>"If income decreases like this, I work a little these days. " - Participant 1
	Season	Winter	"It's easy to fall while working in the winter. And I can't go outside often because it's cold."- Participant 1
		Summer	"Summer is hot, so I usually rest in my yard. I don't work hard in summer" - Participant 1

Broad category	Theme	Subtheme	Exemplary quotes
Inter/action strategies	Work at their own risk	Collection until night	"When shops are closed late at night, they put waste or junk on the driveway in front of the shop. Because of intense competition, many people work at dawn or at night to collect faster. Working on a dark driveway makes traffic accidents easier. - Participant 2<br>"people said my luminous vest was running out of luminous material, because I washed it a lot. I thought it would be safe at night to wear a vest, but it was wrong. I'm worried. I haven't seen a luminous sticker on the rear car. It wasn't usually mine, so I borrowed it from the junk shop" - Participant 2<br>"I've never used luminous vests, never received them." - Participant 3
Inter/action strategies	Work at their own risk	Long time work	"This is making as much money as I collected junk. This work doesn't have any rule of working hours, so if I don't do much, I work for long hours." - Participant 2<br>"There is no fixed working time. I went to collect junk whenever I have some free time. I had to pick up as much as possible, so I worked as long as possible." - Participant 3
		Dangerous collection around driveway	"This(traffic accident) is because we work around the driveway.- Participant 2<br>"When I go to the driveway with my luggage, the cars go by me and I'm too scared"- Participant 4
		Violation of traffic rules	"Because competition is getting more and more intense, we will collect it by jaywalking if we see waste paper or junk on the street." - Participant 2<br>"Here's the road in front of the junk shop. .I have seen traffic accidents many times when old people pulled their luggage cross the road" - Participant 5
		Heavy objects with difficulty in viewing	"Because of junk piled up like mountains in the rear car, we can't see the back of the driveway. There's no rear-view mirror in the rear car. As a result, traffic accidents easily cross the street." - Participant 2
		Carrying heavy load	"If I collect as much as possible, I collect 50kg and go to junk shop. Some men also carry 100kg luggage." - Participant 3
		Poor working posture	"I leaned down, especially when I collected junk," - Participant 2<br>"I carry junk over my head" - Participant 3
	Frustration	Social stigma	"I was accused of picking up trash. Some people disregard me for collecting garbage. The people around the house hate me when I stack the waste paper or junk." - Participant 1<br>"Sometimes some people insult me by telling me to beg."- Participant 2<br>"Someone even quarreled with me for picking up trash."- Participant 3
		Low income	"When I saw it at the beginning of the year, I earned 200,000 won a month. Nowadays, the price of waste paper is so low that I make about 100,000 won a month. When I make so little money,<br>I feel depressed about this work " - Participant 1
Inter/action strategies	Happiness and self-esteem from job	Happiness and self-esteem from job	"I was really depressed when I was only home because I had been sick for 2 years.. However, thesedays, I'm much less depressed and healthy when I'm outside." - Participant 2<br>"I'm not working these days, so I feel lethargic and depressed."- Participant 3<br>"I like this because I can forget everything if I walk around. I work every day without a day off, even Sundays" - Participant 4

Broad category	Theme	Subtheme	Exemplary quotes
Consequences	Occupational injury	Traffic accident	"Fortunately I did not experience accidents. But five years ago, my friend was killed in a car accident on the driveway while collecting junk." - Participant 4
			"There was an accident. When I was picking up junks, the car hit both legs from behind. I was squatting around the road and didn't know if the car was coming." - Participant 3
		Fall	"Then I sometimes lose my balance and I fall." - Participant 3
			"When it snows in winter, the road is slippery, and the bicycle falls easily. It's easy to fall when I'm carrying a heavy load on my bike, going down a slope, or passing a speed bump." - Participant 1
	Musculoskeletal disease	Musculoskeletal disease	"I feel neck and back pain." - Participant 2
			" my shoulder hurts a lot" - Participant 4
	Depression	Depression	"I tried to suicide by eating sleeping pills. I went to a psychiatric hospital for depression." - Participant 4

Table S2. Average hourly energy consumption by occupation.

Occupation	Average hourly energy consumption (kcal)
Recyclable materials collector†	128.5
White collar worker‡	22.9
Car manufacturing worker§	49.4
Form work Carpenter¶	115.2

†2 workers, our study result; ‡3 workers, Labor intensity assessment project, Korea GM, 2016; §29 workers, Labor intensity assessment project, Korea GM, 2016; ¶18 workers, Form carpenter`s labor intensity assessment project, 2018.

Table S3. Risk grade distribution of by elements of work according to REBA.

ID†	Elements of work	Job composition rate (%)	Risk grade distribution according to REBA (%)			
			Normal	Low risk	Moderate risk	High risk
A	Collecting	29.8	0.0	42.9	50.0	7.1
	Carrying	14.9	0.0	42.9	57.1	0.0
	Classifying	14.9	0.0	71.4	28.6	0.0
	Moving	40.4	78.9	21.1	0.0	0.0
	Total	100	31.9	38.3	27.7	2.1
B	Collecting	32.8	9.5	4.8	71.4	14.3
	Carrying	4.7	0.0	66.7	33.3	0.0
	Classifying	26.6	5.9	52.9	41.2	0.0
	Moving	35.9	91.3	8.7	0.0	0.0
	Total	100.0	37.5	21.9	35.9	4.7

†participant identification.

Table S4. The distribution of body position.

Body region	Position description	Participant A Rate (%)	Participant B Rate (%)
Neck	Bent forward 0°~20°	82.8	83.8
	Bent forward over 20°	10.9	5.4
	Bent backward	6.3	10.8
	Twisted / side bending	1.6	0
Upper arm	Bent -20°~20°	48.4	27
	Bent forward 20°~45° / Bent backward over 20°	15.6	27
	Bent forward 45°~90°	31.3	35.1
	Bent forward over 90°	4.7	10.8
	Abducted or rotation	3.1	5.4
	Shoulder raised	3.1	5.4
	arm is supported or person is leaning	4.7	13.5
Lower arm	Bent 60°~100°	62.5	56.8
	Bent 0°~60°	34.4	43.2

	Bent 100° or more positions	3.1	0
Wrist	Bent -15°~15°	64.1	75.7
	Bent over 15° / Bent over -15°	35.9	24.3
	wrist is bent from midline or twisted	0	0
Trunk	Straight	18.8	13.5
	Bended Upper body forward 0°~20°	28.1	43.2
	Bended Upper body backward 0°~20°	0	0
	Bended Upper body forward 20°~60°	35.9	13.5
	Bended Upper body backward over 20°	0	0
	Bended Upper body forward over 60°	17.2	29.7
	Twisted / side bending	0	0
Leg (knee)	Bilateral weight bearing	100	100
	Unilateral weight bearing	0	0
	Bended knee 30°~60°	0	16.2
	Bended knee over 60°	9.4	0
